# Cystic brain metastases radiologically simulating neurocysticercosis

**DOI:** 10.1590/S1516-31802011000500011

**Published:** 2011-09-01

**Authors:** Charlene Troiani, Carla Cristina Barbosa Lopes, Carlos Antônio Scardovelli, Gisele Alborghetti Nai

**Affiliations:** I Undergraduate student. School of Medicine, Universidade do Oeste Paulista (Unoeste), Presidente Prudente, São Paulo, Brazil.; II MD. Intern in the Neurosurgery Service, Hospital Regional de Presidente Prudente, Presidente Prudente, São Paulo, Brazil.; III MD. Neurosurgeon, Hospital Regional de Presidente Prudente, Presidente Prudente, São Paulo, Brazil.; IIII MD, PhD. Professor of Pathology, Department of Pathology, School of Medicine, Universidade do Oeste Paulista (Unoeste), Presidente Prudente, São Paulo, Brazil.

**Keywords:** Neoplasm metastasis, Neurocysticercosis, Cysticercus, Brain diseases, Magnetic resonance imaging, Metástase neoplásica, Neurocisticercose, Cisticerco, Doenças cerebrais, Imagem por ressonância magnética

## Abstract

**CONTEXT:**

Brain metastases are common complications of cancer. Magnetic resonance imaging (MRI), the main diagnostic imaging method in these cases, rarely shows cystic images.

**CASE REPORT:**

The patient was a 45-year-old woman who had had severe headache for a month that was refractory to medication, and had previously had breast cancer, which had been treated. The MRI showed the criteria for neurocysticercosis. Since there was no improvement with clinical treatment, we chose to excise the lesions. Histopathological analysis showed an epithelioid malignant neoplasm.

**CONCLUSION:**

From immunohistochemical analysis, it was concluded that this was a metastasis of breast carcinoma. Even when the MRI is not characteristic of cerebral metastasis, this hypothesis needs to be ruled out in patients with a previous history of cancer.

## RESUMO

**CONTEXTO:**

Metástases cerebrais são complicações comuns do câncer. A ressonância nuclear magnética (RNM), principal método de diagnóstico por imagem nesses casos, mostra raramente imagens císticas.

**RELATO DO CASO:**

A paciente era uma mulher de 45 anos de idade, com cefaleia intensa há um mês, refratária à medicação, e câncer de mama tratado previamente. A RNM apresentava critérios diagnósticos para neurocisticercose. Como não apresentou melhora com tratamento clínico, optou-se pela exérese das lesões. O histopatológico mostrou neoplasia maligna epitelioide.

**CONCLUSÃO:**

A imunoistoquímica revelou metástase de carcinoma da mama. Mesmo quando a RNM não é característica de metástase cerebral, esta hipótese precisa ser afastada em pacientes com história prévia de câncer.

## INTRODUCTION

Brain metastases are common complications of cancer, and show increasing incidence. They may present clinically as headache, seizures or loss of cognitive or motor function depending on the location of the lesions.^1^ Cancers of the breast, lung, kidney, gastrointestinal tract and skin are the commonest causes of cerebral metastases.^1^

Magnetic resonance imaging (MRI) is the main diagnostic imaging method. However, metastases rarely show multiple cystic images on MRI.^2^

The World Health Organization (WHO) considers that neurocysticercosis is the commonest parasitic disease of the brain; its main manifestation is seizure.^3,4^ The prevalence of neurocysticercosis in Brazil is unknown because of the absence of mandatory reporting in most states. The seropositivity reaction rate in Brazil is 2.3%, representing 0.3% of all hospitalizations.^5^ Neurocysticercosis has been reported in most Brazilian states, with highest prevalence in Paraná, São Paulo, Minas Gerais and Goiás. The costs due to neurocysticercosis treatment and complications in Brazil may reach up to US$ 85 million.^6^

We report an unusual case of cystic brain lesions with a radiological diagnosis of neurocysticercosis, but a pathological diagnosis of brain metastases. This case shows the importance of valuing the patient's clinical history in the differential diagnosis of multiple brain lesions.

## CASE REPORT

A 45-year-old female was admitted to the Regional Hospital in Presidente Prudente, State of São Paulo, with a complaint of severe headache for a month that was refractory to medication. She had a history of breast cancer that had been treated with radiotherapy three years before the time of the admission described here.

**Figure 1:**
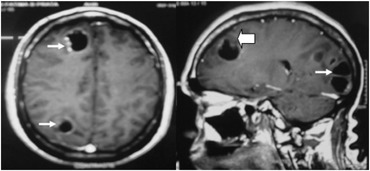
Magnetic resonance imaging (MRI) showing rounded cysts characterized by low signal on T1-weighted sequences and hyperintense on T2-weighted sequences (thin arrows). Some cysts have small nodules on their wall (large arrow). Note the contrast enhancement on the periphery of the cyst and more intensely in the region of mural nodules.

Computed tomography (CT) showed several hypodense lesions in cerebral parenchyma. MRI showed cystic lesions with an internal nodular structure that was similar to a scolex, thus suggesting the diagnosis of neurocysticercosis. The lesions also had contrast enhancement on the periphery of the cyst that was more intense in the region of mural nodules (Figure 1).

**Figure 2:**
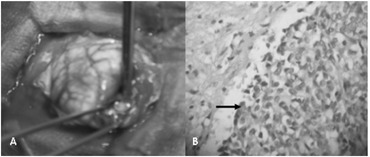
(A) Surgical resection of brain lesion. (B) Optical microscopy showing epithelioid malignant neoplasm (arrow) (hematoxylin and eosin, 250 x).

The patient was treated with albendazole for five days, but without improvement. She then underwent surgery to resect the lesions (Figure 2A).

The pathological examination revealed an epithelioid malignant neoplasm (Figure 2B). Immunohistochemical analysis showed positivity for pan-cytokeratin markers (AE1/AE3) and cytokeratin 7 (CK7) and negativity for cytokeratin 20 (CK20), S-100 protein, estrogen receptor and thyroid transcription factor-1 (TTF-1). From this, the conclusion was a diagnosis of metastatic breast cancer.

The patient underwent radiotherapy, but without improvement. She was admitted to the hospital sometimes with severe headache and vomiting and cerebral edema, but missed the follow-up after one year.

## DISCUSSION

Cysts are common findings on MRI and CT brain imaging. Their histopathological spectrum is broad, and differentiation of these cysts on the basis of imaging findings alone can be problematic.^7,8^

Neurocysticercosis is the most common parasitic disease of the central nervous system (CNS) and should be considered to be a differential diagnosis of brain metastases, especially in patients in endemic countries, because of the similarity of clinical manifestations.^9^ We searched in the Li­lacs (Literatura Latino-Americana e do Caribe em Ciências da Saúde), IBECS (Índice Bibliográfico Espanhol de Ciências de Saúde), Scirus, Embase (Excerpta Medica database) and Medline databases, using MeSH (Medical Subject Headings), and only one case report related to the differential diagnosis between neurocysticercosis and brain metastasis^9^ (Table 1).

**Table 1:** Results from the review of the medical databases, using descriptors relating to the differential diagnosis of this case

Database	Search strategy	Results
Medline	"Neurocysticercosis"	374 case reports
		95 retrospective studies
		72 incidence studies
		56 prevalence studies
		12 cohort studies
		37 case-control studies
		3 editorials/communications
		5 narrative reviews
Lilacs		66 case reports
		134 retrospective studies
		19 incidence studies
		19 prevalence studies
		10 case-control studies
		6 randomized controlled trials
		10 narrative reviews
IBECS		10 case reports
		41 retrospective studies
		1 incidence study
		1 prevalence study
		2 narrative reviews
Scirus		1242 case reports
		297 retrospective studies
		149 incidence studies
		134 prevalence studies
		17 cohort studies
		43 case-control studies
		7 editorials/communications
		13 narrative reviews
Embase		69 case reports
		153 retrospective studies
		117 letters
		17 editorials/communications
		135 narrative reviews
Medline	"Neurocysticercosis" and "brain metastasis"*	1 case report (Coulibaly et al.^9^)
		2 retrospective studies
		1 incidence study
		1 case-control study
Scirus		34 case reports
		39 retrospective studies
		10 incidence studies
		7 prevalence studies
		5 case-control studies
		3 narrative reviews
Embase		4 retrospective studies
		1 letter
Medline	"Brain metastasis" and "cyst"†	10 case reports
		77 retrospective studies
		1 incidence study
		5 narrative reviews
Lilacs		1 case report
		1 review
Scirus		1751 case reports
		430 retrospective studies
		297 incidence studies
		238 prevalence studies
		39 cohort studies
		72 case-control studies
		27 narrative reviews
Embase		1 case report
		1 retrospective study
Medline	"Brain metastasis" and "breast cancer"	217 case reports
		336 retrospective studies
		70 incidence studies
		6 prevalence studies
		20 cohort studies
		8 case-control studies
		10 narrative reviews
Lilacs		5 case reports
		4 retrospective studies
		2 incidence studies
IBECS		1 case report
		2 retrospective studies
Scirus		9564 case reports
		2244 retrospective studies
		1692 incidence studies
		1308 prevalence studies
		572 cohort studies
		329 case-control studies
		8 editorials/communications
		135 narrative reviews
Embase		90 case reports
		210 retrospective studies
		56 incidence studies
		51 cohort studies
		39 case-control studies
		17 letters
		4 editorials/communications
		68 narrative reviews

* Using the same search strategy in Lilacs and IBECS database, no results were found;

† Using the same search strategy in IBECS database, no results were found.

Neurocysticercosis occurs in 60% to 90% of all cases of systemic cysticercosis.^7^ A typical presentation of cerebral cysticercosis may mimic glioma, metastasis or cerebral abscess, or vice versa.^10^ Most neurocysticercosis cysts are found in the subarachnoid spaces: typically the basal cisterns and deep within the sulci.^7^ A smooth thin-walled cyst on MRI images typifies the early vesicular stage of neurocysticercosis. Edema and contrast enhancement are rare. A mural nodule is often present that represents the viable larval scolex, i.e. the "cyst with a dot" appearance.^3,7,9^ Usually, the lesions are < 20 mm in diameter.^11^ Identification of brain vesicles with scolex is pathognomonic of neurocysticercosis, while inflammatory changes around the lesion indicate the degenerative process of the parasite.^2^ About 15% of patients with neurocysticercosis have a unique cysticercus in the CNS.^3^ In the case reported here, the multiple lesion-like "cyst with a dot" appearance on MRI suggested the diagnosis of neurocysticercosis. One clue in this case could be the contrast enhancement around all the lesions, which is not seen in neurocysticercosis but occurs in brain metastases. Even in countries where neurocistecercosis is endemic and MRI abnormalities were suggestive of scolex, brain metastasis must be ruled out in the presence of contrast enhancement around the lesions, especially in patients with previous history of cancer.

Establishing the diagnosis of a brain tumor is not always a straightforward process. Many neoplastic neurological diseases can mimic brain neoplasms on neuroimaging or on histological examination, including multiple sclerosis, stroke, pyogenic abscess, toxoplasmosis, tuberculosis, cysticercosis, fungal infection, syphilis, sarcoidosis, Behçet disease, radiation necrosis, venous thrombosis, and others.^12^

Primary brain tumors are rarely multiple. Pilocytic astrocytoma, ganglion cell tumors, ependymomas, hemangioblastomas and pleomorphic xanthoastrocytoma are some of the primary brain tumors that can be cystic. Of these, hemangioblastomas may be epithelioid.^1^

To determine whether a tumor is primary or not, and the primary site of metastatic tumors, immunohistochemical analysis can be used, with specific markers for each tissue and cell.^6^ The use of multiple markers may increase the sensitivity, specificity and positive predictive value in predicting the tissue of tumor origin.^13^

Metastatic adenocarcinoma of unknown primary site is a common clinical problem. The use of cytokeratin 20 (CK20) and 7 (CK7) can assist in identifying primary sites in this situation. CK20 positivity indicates only adenocarcinoma metastasis of many organs. CK7 negativity is consistent with metastatic adenocarcinoma of the lung, ovaries, liver or serous membranes.^14^

Astrocytic neoplasms and hemangioblastomas (neoplasms of vascular origin) do not test positive for cytokeratin (epithelial cell marker). Negativity for the TTF-1 marker rules out the possibility of thyroid and primary neuroendocrine lung tumor as the primary site. Cytokeratin 7 positivity and cytokeratin 20 negativity promote the breast as the primary site. Negativity for estrogen receptors does not rule out this possibility, since many breast tumors are not positive for hormonal receptors,^15^ as occurred in this case.

In brain metastases, intracranial hypertension and motor deficits are common. The most sensitive methods for diagnosis are CT and MRI when there is no meningeal involvement. In these cases, examination of cerebrospinal fluid (CSF) may be more effective. The use of tumor markers such as CEA (carcinoembryonic antigen) and CA-15.3 (often elevated in breast carcinomas) in routine CSF examinations has shown promising results, especially in relation to leptomeningeal metastases.^16^

The survival with a single brain metastasis is longer than with multiple brain metastases, and hence the treatment in such cases consists of immediate surgery, when feasible, and optimal adjuvant therapy.^17^ In our case, although the patient has undergone radiotherapy, there was no clinical improvement, probably due to the presence of multiple brain lesions.

Distinguishing non-neoplastic causes from neoplastic lesions is extremely important, because misdiagnosis can lead to unwarranted neurosurgery and exposure to toxic chemotherapy or potentially harmful brain irradiation. Diligent clinical evaluation and a battery of tests are required, for a definitive diagnosis to be made.^11^ Newer advanced diagnostic techniques, such as diffusion-weighted MRI, perfusion-weighted MRI, magnetic resonance spectroscopy, single-photon emission tomography and positron emission tomography, as well as new tools for histological examination, such as immunohistochemical and molecular genetic analysis can help in establishing the diagnosis.^11,12,18-20^

## CONCLUSION

Even in cases in which MRI is not characteristic of cerebral metastasis, this hypothesis needs to be excluded when there is no improvement with clinical treatment and in patients with a previous history of cancer, especially breast cancer, which is a common cause of brain metastasis.
